# Effect of follicle size on oocytes recovery rate, quality, and *in-vitro* developmental competence in *Bos indicus* cows

**DOI:** 10.1590/1984-3143-AR2020-0011

**Published:** 2020-09-09

**Authors:** Zaeem Sarwar, Muhammad Saad, Muhammad Saleem, Ali Husnain, Amjad Riaz, Nasim Ahmad

**Affiliations:** 1 Department of Theriogenology, Faculty of Veterinary Science, University of Veterinary and Animal Sciences, Lahore, Pakistan; 2 Department of Theriogenology, Faculty of Veterinary Science, Cholistan University of Veterinary and Animal Sciences, Bahawalpur, Pakistan; 3 Department of Animal Sciences, University of Florida, Gainesville, FL, United States

**Keywords:** Size of follicle, *in-vitro* developmental competence, *Bos indicus* cows

## Abstract

The objective of the present study was to determine the effect of follicle size on recovery rate, quality, and *in-vitro* developmental competence of oocytes in *Bos indicus* cows. The ovaries (n = 507) of *Bos indicus* cows having age of 5-8 years, with mixed parity, BCS 2.75 ± 0.25, and clinically normal reproductive tracts were collected from the local abattoir. The follicles on the ovaries were divided into two groups based upon their size; 1) ≥6 mm diameter, and 2) <6 mm diameter. After initial evaluation of quality of the oocytes, the COCs were *in vitro* matured, fertilized, and cultured to determine the *in vitro* developmental competence. The oocyte recovery, quality, maturation, cleavage, 4-cell, 8-cell, and 16-cell stages were analyzed using PROC GLIMMIX procedure of SAS. However, the number of oocytes recovered per ovary was analyzed using MIXED procedure of SAS. Results revealed that the recovery of oocytes (LSM ± SEM) derived from the follicles having size <6 mm per ovary was greater (1.02 vs. 3.14 ± 0.13; *P* < 0. 0001). However, the percentage (n/n) recovery [69.8 (474/679) vs. 62.7% (1454/2320); *P* = 0.01] and grade I_+_II oocytes [68.4 (324/474) vs. 57.9% (842/1454); *P* < 0.0001] was greater in ≥6 mm as compared with <6 mm group, respectively. However, maturation rate did not differ [92.9 (288/310) vs. 92.2% (296/321); *P* = 0.98] between the groups. In contrast, cleavage rate [58.1 (180/310) vs. 47.4% (152/321); *P* = 0.01], the 4-cell [34.5 (107/310) vs. 18.7% (60/321); *P* = 0.0003], 8-cell [15.5 (48/310) vs. 7.8% (25/321); *P* = 0.008], and 16-cell [8.7 (27/310) vs. 2.1% (7/321); *P* = 0.004] stage embryos were greater in ≥6 mm group. It can be concluded that oocytes derived from follicle ≥6 mm have better *in vitro* developmental competence based on embryonic conversion in *Bos indicus* cows.

## Introduction

Assisted reproductive technologies (ART) have revolutionized the dairy industry in the past century for the rapid multiplication and up gradation of the superior genetics (Moore and Hasler, 2017). However, genetic exploitation through *in-vitro* embryo production (IVEP) in the farm animals remained challenging during the last three decades (Thompson, 1997) The IVEP involves oocyte collection, maturation, sperm capacitation, fertilization, and embryo culture (Rose and Bavister, 1992). Several factors influence the outcome of IVEP technique which include, oocytes obtained either from slaughtered or live animal (Gasparrini, 2002), parity (Snijders et al., 2000a), age (Armstrong, 2001), stage of the estrous cycle (De Wit et al., 2000), blood progesterone concentration (Saad et al., 2019), body condition score (BCS), milk production (Snijders et al., 2000b), season of the year (Al-Katanani et al., 2002), cyclicity (Boediono et al., 1995), capacitation method (Parrish et al., 1995), sex preselection of embryos (Chowdhury et al., 2019) and size of the follicle (Pavlok et al., 1992; Lonergan et al., 1994). These factors are in addition to the laboratory procedures (Gordon, 2003) which are the key determinants for the success of IVEP technique.

Under *in vivo* conditions about 85% embryos were developed from the spontaneously ovulated oocytes in normal cyclic cows (Montalenti, 1968). In contrast, during IVEP only about 20-40% transferable embryos were produced from oocytes derived from slaughterhouse ovaries or ovum pick up (Pavlok et al., 1992; Lonergan et al., 1994; Otoi et al., 1997; Lequarre et al., 2005; Camargo et al., 2018). The difference is because oocytes resuming meiosis *in vivo,* originate from the follicles having size >15 mm. Moreover, *in vivo* derived oocytes mature in approximately five days and achieve a size of >15 mm (Driancourt, 1991). Contrarily, the oocytes for *in vitro* studies are derived from different sized follicles varying from 2-15 mm and matured only for 24 hours. Due to which the meiosis resumption abilities of oocytes varies *in vitro* (Pavlok et al., 1992; Lonergan et al., 1994; Otoi et al., 1997; Lequarre et al., 2005; Caixeta et al., 2009) Furthermore, the capabilities of the oocytes to fertilize and grow *in vitro* up to advanced embryonic stages have been known to be dependent upon the size of follicle from which oocytes were retrieved. The oocytes recovered from ≥6 mm follicles were better in terms of quality and *in vitro* developmental competence (Pavlok et al., 1992; Lonergan et al., 1994) Variations in the size of follicle lead to the difference in the *in vitro* developmental competencies of the oocytes (Hendriksen et al., 2000). Thus, the size of the follicles from which the oocytes are retrieved is a key factor to determine IVEP rates along with other factors (Pavlok et al., 1992; Lonergan et al., 1994; Crozet et al., 1995; Caixeta et al., 2009). The mechanism why blastocyst yield is lower in small follicles is because they have delayed cavitation and retorted growth stages of cell cycle (Lequarre et al., 2005). Therefore, we hypothesized that oocytes recovery, quality and *in vitro* developmental competence in ≥6 mm sized follicles will be better than the <6 mm sized follicles in *Bos indicus* cows.

*Bos indicus* cattle are best known for heat tolerance and tick resistance in tropical and subtropical regions (Glass et al., 2005; Khan et al., 2008). The reproductive physiology of *Bos taurus* is quite different from *Bos indicus* cows (Sartori et al., 2010). *Bos indicus* cows require longer times to attain puberty, have prolonged postpartum anestrus duration, shows greater tendency of seasonal breeding, and displays a shorter duration of estrus with fewer overt signs (Bó et al., 2003). Most of the work on ART has been conducted in *Bos taurus.* Similar information is generally lacking in *Bos indicus* cows. It was hypothesized that for the oocytes recovered from the follicles having size ≥6 mm will have greater recovery rate, oocytes quality as well as an early *in vitro* developmental competence as compared to the oocytes derived from <6 mm follicles. Therefore, the objective of the present study was to determine the effect of follicle size on recovery rate, quality, *in-vitro* developmental competence of oocytes based on embryonic conversion in *Bos indicus* cows.

## Materials and methods

This study was conducted during 2018 in the embryology laboratory of the University of Veterinary and Animal Sciences, Ravi Campus, Pattoki, Punjab-Pakistan. The research was approved by the Ethical Review Committee of UVAS, Lahore (Reference No. DR/178). All the experiments were performed using the commercially available IVF media (Minitüb GmbH, Germany) and all the reagents of Sigma Chemical Company (St. Louis, MO, USA).

### Collection of oocytes and experimental design

The *Bos indicus* ovaries (n = 507) were collected from slaughtered cows having 5 - 8 years of age and mixed parity, with clinically normal reproductive tracts. The ovaries were brought to laboratory in solution containing 0.9% sodium chloride solution (Sigma; cat # S5886), added with 50 mg/ml streptomycin (Sigma; cat # P4562) and 500 µg/ml penicillin (Sigma; cat # P3032) and maintained at temperature of 30-35^o^C within 2 hours after slaughtering. Later, in the laboratory, the ovaries were washed three times with normal saline in to minimize the contamination.

The follicles were classified based upon their size into two groups: 1) ≥6 mm, and 2) <6 mm as classified by (Lonergan et al., 1994). Afterwards, oocytes were aspirated with 18 G needle and 10 ml syringe by ovum pick up media (Minitüb GmbH, Germany). The cumulus-oocyte complexes (COCs) were categorized into four classes based on the number of COCs layers and cytoplasmic aspects (Kastrop et al., 1990). Only Grade I_+_II oocytes were selected for advanced processing. Thereafter, COCs were washed three times in TL HEPES solution (Minitüb GmbH, Germany) supplemented with 6 mg/ml bovine serum albumin (BSA; Sigma fraction V, cat # A6003).

### In vitro maturation (IVM)

Oocytes were washed three times in IVM medium consisting of (TCM 199 stock solution; Minitüb GmbH, Germany) added with estrus cow serum (OCS, own produced and deactivated), 0.5 µg/ml, follicle stimulating hormone (FSH; Sigma; cat # F2293) and 0.25 µg/ml, luteinizing hormone (LH; Sigma; cat # L5269). All COCs of the two groups (≥6 mm vs. <6 mm) were placed in separate 100 µl IVM drops in four well plates (SPL Life Sciences, Korea) covered with a pre-warmed mineral oil layer (Sigma-M5310). Four well plates were incubated for a period of 24 hours in CO_2_ incubator (Shel lab; 3503-2, USA) with 5% CO_2_ and with maximum humidity at 38.5^o^C. After 24 hours, IVM was evaluated based on degree of expansion of the COC layers and on the success of extrusion of the first polar body (Pavlok et al., 1992).

### In vitro fertilization (IVF)

After the IVM, the COCs were washed three times in the drops of IVF media consisted of TL stock solution (Minitüb GmbH, Germany) supplemented with BSA (6mg/ml), sodium-pyruvate (Sigma; cat # P4562; 10 µl/ml), and heparin (Sigma; cat# H3149; 20 µl/ ml). After washing, COCs were placed in 100µl IVF media droplets covered with pre warmed mineral oil (Sigma-M5310). The capacitated semen (50 µl/ drop) was added into the IVF drops and four well plates were incubated for a time period of 18 hours at 38.5^o^ C, 5% CO_2_ along with maximum relative humidity.

For *in vitro* capacitation of the sperms two straws of progeny tested Sahiwal bull (*Bos indicus*) having the same batch and of known fertility were pooled into the falcon tube. The semen having >40% post thaw motility was placed at the bottom of capacitation media. This media comprised of TL stock solution (Minitüb GmbH, Germany) supplemented with BSA (6 mg/ml), 50 µl/ml sodium-pyruvate (Sigma; cat # P4562), and 1 µl/ml gentamicin (Sigma; cat # 345815). Thereafter, the falcon tube was incubated at 38.5^o^C for 30 minutes in tilted form. Furthermore, motile sperms from supernatant were transferred and centrifuged at 328g for 10 minutes in another sterile falcon tube as described earlier (Pavlok et al., 1992). After discarding supernatant, the remaining pellet was mixed gently with capacitation media to make the final concentration of 2 million sperms per ml.

After 18 hours, fertilization was evaluated based on presence of male and female pronuclei or cleavage (Lonergan et al., 1994).

### In vitro culture (IVC)

After fertilization evaluation, the presumptive zygotes were washed three times in media containing TL HEPES (Minitüb GmbH, Germany) solution supplemented with BSA (6 mg/ml). Meanwhile, denuding of zygotes was performed with gentle pipetting. Subsequently, presumptive zygotes were washed thrice in IVC media droplets composed of SOF stock solution (Minitüb GmbH, Germany) supplemented with 1 ml estrus cow serum (own produced and deactivated), 40 µl/ml amino acids essential (Sigma; cat # M5550), and 10 µl/ml amino acids non-essential (Sigma; cat # M7145). After washing, the presumptive zygotes were placed in pre-warmed 100µl IVC droplets, overlaid with mineral oil at 38.5^o^C, 5% CO2 and 95% relative humidity. To check every cell stage, *In vitro* embryonic development was recorded on days 3, 4, 5, and 6 after the day of insemination.

### Statistical analysis

Continuous response variable that is the mean number of oocytes recovered per ovary from follicles having size ≥ 6mm and < 6mm was analyzed by using the MIXED procedure of SAS (SAS ver. 9.4 Institute, Inc., Cary, NC, USA). The proportion of the oocytes recovered per follicle, oocyte quality, maturation, cleavage, embryo development to the 4-cell, 8-cell, and 16 cell stages when oocytes were derived from either ≥ 6mm and < 6mm oocytes were analyzed using the PROC GLIMMIX procedure of SAS (SAS ver. 9.4 Institute, Inc., Cary, NC, USA). The data were of binominal distribution. All models included the fixed effect of follicle size and the random effect of replicate (day of collection of ovaries). The contemporary group on a specific slaughter day was considered as the experimental unit. The level of significance to reject the null hypotheses (HO) was 5%, and values for a variable were different when *P* ≤ 0.05.

## Results

Effect of follicle size on the percent oocytes recovery, quality of oocytes and *in vitro* developmental competence of the embryos in *Bos indicus* cows has been presented in the Table 1. The odds of recovery of oocytes for follicle ≥6 mm was 28% greater (Odds ratio; 1.28, 95% CI; 1.06-1.56, *P* = 0.01) than the odds for follicle <6 mm. The percentage of grade I_+_II oocytes was greater [68.4 (324/474) vs. 57.9% (842/1454); *P* < 0.0001] and the oocytes with grade III_+_IV were lower [31.6 (150/474) vs. 57.9% (842/1454); *P* < 0.0001] between the oocytes recovered from the follicles having size ≥6 mm as compare with <6 mm. However, the maturation rate did not differ (Odds ratio; 1.01, 95% CI; 0.54-1.88, *P* = 0.98) between the groups. The *in-vitro* developmental competence of oocytes revealed that the cleavage rate [58.1 (180/310) vs. 47.4% (152/321); *P* = 0.01], 4-cell stage [34.5 (107/310) vs. 18.7% (60/321); *P* = 0.0003] and 8-cell stage embryos [15.5 (48/310) vs. 7.8% (25/321); *P* = 0.008] were greater in the oocytes obtained from ≥6 mm sized follicles as compared with the oocytes recovered from <6 mm follicle. Similarly, the 16-cell stage embryos were greater [8.7 (27/310) vs. 2.1% (7/321); *P* = 0.004] in ≥6 mm group. Results revealed that oocytes recovered from follicles having size ≥6 mm have better recovery, quality and *in vitro* developmental competency based on embryonic conversion in *Bos indicus* cows.

**Table 1:** Percentage (n/n) along odds ratio with 95% confidence interval for the effect of follicle size on recovery, quality, and *in-vitro* developmental competence of oocytes in *Bos indicus* cows^1^.

**Variables**	**≥ 6mm**	**< 6mm**	**Odds ratio**	**95% confidence interval**		***P*-value**
Oocyte recovery per follicle2; %	69.8 (474/679)	62.7 (1454/2320)	1.28	1.06	1.56	0.01
Grade I_ +_ II oocyte3; %	68.4 (324/474)	57.9 (842/1454)	3.04	2.40	3.85	<0.0001
Grade III_+_IV oocyte4; %	31.6 (150/474)	57.9 (842/1454)	0.33	0.26	0.42	<0.0001
Matured oocyte5; %	92.9 (288/310)	92.2 (296/321)	1.01	0.54	1.88	0.98
Cleavage6; %	58.1 (180/310)	47.4 (152/321)	1.54	1.1	2.15	0.01
4-cell stage embryo7; %	34.5 (107/310)	18.7 (60/321)	2.29	1.54	3.39	0.0003
8-cell stage embryo8; %	15.5 (48/310)	7.8 (25/321)	2.21	1.27	3.84	0.008
16-cell stage embryo9; %	8.7 (27/310)	2.1 (7/321)	4.14	1.77	10.30	0.004

1 Oocytes were categorized based upon the size of follicle ≥ 6mm or < 6mm. ^2^ For odds ratio the reference was the oocyte derived from the size of follicle < 6mm. ^3^Grade I_+_II oocyte; number of oocytes with grade I_+_II/ total number of oocytes recovered٭100. ^4^Grade III_+_IV oocyte; number of oocytes with grade III_+_IV/ total number of oocytes recovered٭100. ^5^Matured oocyte; number of oocytes matured/ total number of oocytes cultured for in vitro maturation٭100. ^6^Cleavage; number of oocytes cleaved/ total number of oocytes cultured for in vitro maturation٭100. ^7^4-cell stage embryo; number of 4-cell stage embryos/ total number of oocytes cultured٭100. ^8^8-cell stage embryo; number of 8-cell stage embryos/ total number of oocytes cultured٭100. ^9^16-cell stage embryo; number of 16-cell stage embryos/ total number of oocytes cultured٭100.

**Figure 1:**
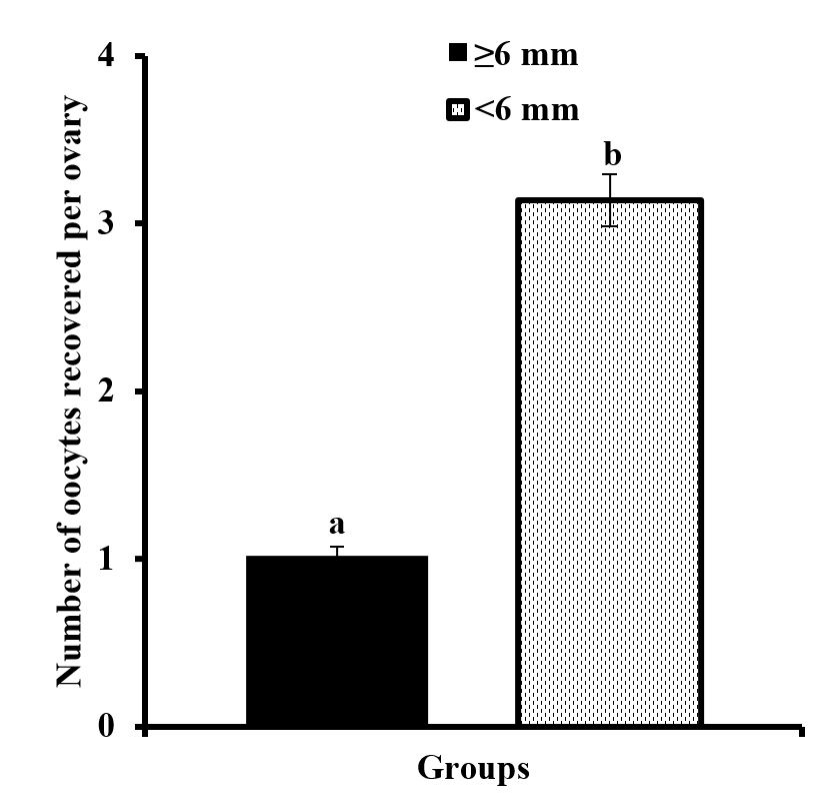
Effect of follicle size on number of oocytes recovered per ovary (least square mean ± SEM) from ≥6 mm and <6 mm groups in *Bos indicus* cows. ^a,b^ superscripts differ (*P* < 0.05) between both groups.

Effect of size of follicle on oocytes recovered per ovary (LSM ± SEM) in *Bos indicus* cows is presented in Figure 1. Greater number of follicles having size <6 mm were present per ovary in *Bos indicus* cows. Therefore, greater (1.02 vs. 3.14 ± 0.13, *P* < 0.0001) was the recovery of oocytes per ovary from follicles size <6 mm as compared with ≥6 mm sized follicle.

## Discussion

Present study determines the effect of follicle size on the recovery rate, quality and *in vitro* developmental competence of oocytes in *Bos indicus* cows. We hypothesized that ≥6 mm follicles would have better recovery, quality, and *in-vitro* developmental competency of oocytes (Pavlok et al., 1992; Lonergan et al., 1994). In the present study, the most salient findings were manifested that the cleavage rate, number of 4-cell and 8-cell stage embryos were greater in the oocytes recovered from ≥6 mm (larger follicles) as compared with <6 mm follicles (smaller follicles), respectively. Likewise, the 16 cell stage embryos (16-cell) were also greater from the oocytes retrieved from the large follicles. These findings are supported by the previous studies, in which the oocytes derived from ≥6 mm follicles for IVEP yielded better quality embryos in *Bos taurus* cows (Pavlok et al., 1992; Lonergan et al., 1994; Lequarre et al., 2005).

The most plausible reasons for the lower IVEP rates of oocytes derived from <6 mm follicles includes incomplete nuclear and cytoplasmic maturation, greater degree of atresia, (Pavlok et al., 1988), and higher degree of fertilization abnormalities (Pavlok et al., 1988) than the oocytes derived from ≥6 mm follicles. Furthermore, the oocytes derived from <6 mm follicles have inadequate synthesis of maternal mRNAs that are required for the compaction and differentiation of the embryos at advanced stages (Crozet et al., 1986; Shabankareh et al., 2015 and lack of production of essential proteins which are required for *in vitro* growth competence (Sirard et al., 1989). In addition, the previous studies had reported that the oocytes from small follicles have irregular cortical granules distribution, defective system of exocytosis, have greater fertilization abnormalities and thus not capable of supporting the embryonic development (Cran and Cheng, 1986; Hyttel et al., 1986; Shabankareh et al., 2015). Recent evidence had revealed that certain genes like H2A, FSHR, GHR etc. were related to the oocytes competence. But H2A gene and the oocytes specific histone are essentially related to the size of the follicles. The oocytes recovered from<6 mm sized follicles had lower levels of H2A transcripts and oocytes specific histone. Therefore, their developmental competence of the oocytes was compromised (Caixeta et al., 2009) and have the delayed cellular developmental stages of embryos (Lequarre et al., 2005). These are some most plausible reasons for greater *in vitro* developmental competence of the oocytes retrieved from follicles having size ≥6 mm in *Bos taurus* cows. Similar studies are warranted to elucidate the difference of cellular growth, cell cycles and genomic expression between the oocytes retrieved from large and small sized follicles in *Bos indicus* cows.

The results of the present study demonstrated that the recovery of oocytes was greater (*P* < 0.05) in ≥6 mm as compared to <6 mm group, respectively. These results are supported by the previous studies in which the recovery rate was greater from the follicles having size ≥6 mm (Lonergan et al., 1994; Crozet et al., 1995). Likewise, the quality of grade I_ and _II oocytes was about 26% superior for oocytes which were derived from ≥6 mm follicles as compared to <6 mm follicles. Equally, the percentage of good quality oocytes was greater in ≥6 mm group (Lonergan et al., 1994). Moreover, it has been reported that not only the size of follicle, but the diameter of the retrieved oocyte is also positively associated with the enhanced quality and *in vitro* developmental competence of oocytes. The oocytes recovered from larger follicles have greater diameter. Similarly, the oocytes having greater diameter (>115 µm) maximally reach up to the MII and further developmental stages (Otoi et al., 1997).

Previously, it was reported that under *in vivo* conditions, the follicle deviation occurs at a size of 5.4 to 6.1 mm *Bos indicus* cows (Sartorelli et al., 2005; Castilho et al., 2007; Gimenes et al., 2008) and the diameter of the follicle from which ovulation occurred at the time of second GnRH of FTAI protocol influenced the synchronization of ovulation time and pregnancy per AI (P/AI) in cows (Perry et al., 2005). The presence of larger follicle on the day of FTAI improved ovulation rate and P/AI. However, the presence of < 7.5mm follicles on the day of FTAI resulted in reduced ovulation and P/AI (Sá Filho et al., 2010). The most plausible reason is that during follicular growth and oocyte development, mRNA and proteins are produced and stored in the oocyte (Brevini-Gandolfi and Gandolfi, 2001) and developmental competence continues to increase with increased follicular diameter in cows (Arlotto et al., 1996). Similarly, for IVEP, the oocytes obtained from smaller follicles (<6 mm) have lower developmental competence due to incomplete nuclear and cytoplasmic maturation, greater degree of atresia (Pavlok et al., 1988), lack of production of essential proteins that are required for *in vitro* growth competence (Sirard et al., 1989). Therefore, it is plausible to suggest that for good quality oocytes and better outcome of IVEP ≥ 6mm follicles should be aspirated. Therefore, future studies should be done to enhance the size of follicle ≥6mm by using hormonal treatments like FSH and eCG prior to ovum pick up to enhance embryo production rates. And, on the addition of different hormones in IVM media for the oocytes derived from < 6mm follicles.

On ovary, the mean number of ≥6mm follicles was 3 times lower than that of <6 mm follicles. The reason behind this is that, the *Bos indicus* cows have been reported to have smaller ovaries (Mutiga et al., 1993) and higher number of follicles having <5 mm diameter (Fair et al., 1995; Baruselli et al., 2007; Caixeta et al., 2009) than *Bos taurus* cows. The antral follicle population is positively correlated with the plasma concentrations of anti mullerian hormone (AMH). The circulating concentration of AMH is greater in *Bos indicus* as compared to *Bos taurus* cows (Batista et al., 2014). Therefore, the population of follicles on ovaries is greater in *Bos indicus* (Fair et al., 1995; Baruselli et al., 2007) as compared to *Bos taurus* cows.

## Conclusion

The competence of the oocytes was tested based on the ability of embryonic conversion. It was concluded that recovery rate of oocytes, cleavage rate, 4-cell, 8-cell, and 16-cell stage embryos were greater in the oocytes recovered from ≥6 mm as compared to <6 mm follicles in *Bos indicus* cows. It is plausible to suggest that for IVEP ≥6mm follicles should be preferred for aspiration in *Bos indicus* cows.
